# Evaluating an integrated nutrition and mathematics curriculum: primary school teachers’ and students’ experiences

**DOI:** 10.1017/S1368980022000386

**Published:** 2022-08

**Authors:** Berit M Follong, Elena Prieto-Rodriguez, Andrew Miller, Clare E Collins, Tamara Bucher

**Affiliations:** 1 School of Health Sciences, College of Health, Medicine and Wellbeing, The University of Newcastle, University Drive, Callaghan, NSW 2308, Australia; 2 Priority Research Centre for Physical Activity and Nutrition, The University of Newcastle, Callaghan, NSW, Australia; 3 Priority Research Centre for Health Behaviour, The University of Newcastle, Callaghan, NSW, Australia; 4 Teachers and Teaching Centre, School of Education, College of Human and Social Futures, The University of Newcastle, Callaghan, NSW, Australia; 5 School of Environmental and Life Sciences, College of Engineering, Science and Environment, The University of Newcastle, Ourimbah, NSW, Australia

**Keywords:** Healthy eating, Education, Multidisciplinary teaching, Qualitative research, Evaluation

## Abstract

**Objective:**

To present the process evaluation of a curricular Cross-curricular Unit on Portion Size (CUPS) program that integrates nutrition and mathematics, describing teacher and student perspectives on the intervention.

**Design:**

Semi-structured interviews and focus groups were conducted following the implementation of the CUPS program during a pilot randomised controlled trial designed to evaluate efficacy for improved portion size estimation. Lessons involved experiential learning using food models and mathematics cubes and focussed on portion size, food groups, volume and capacity. Data were collected immediately post-intervention and analysed using an inductive thematic approach.

**Setting:**

Primary schools in Newcastle, Australia.

**Participants:**

Year 3 and/or 4 teachers (*n* 3) and their students (*n* 15).

**Results:**

Teachers believed the programme supported the learning of nutrition concepts, with the majority of students enjoying the lessons, cubes and food models. Teachers indicated most students were engaged and became more aware of healthy eating and serve size recommendation. Although teachers enjoyed and valued the lessons, they suggested that the integration of volume and capacity should be further improved in order to address the time barrier for teaching nutrition.

**Conclusion:**

The process evaluation reports on challenges and successes of implementing an integrative nutrition programme. This teaching approach could be useful and successful when aligned with teacher’ and student’ needs. Based on participant feedback, lessons could be refined to enhance integration of mathematics content and to support student learning.

Childhood overweight and obesity presents an ongoing public health concern^(1)^ with an extensive number of prevention interventions, including school-based nutrition interventions, proposed to improve children’s dietary behaviours and prevent childhood obesity^(2,3)^. Previous reviews have demonstrated school-based nutrition education programmes to be moderately effective in reducing the prevalence of overweight and obesity^(4)^, lowering energy intake^(5)^, increasing fruit and vegetable consumption^(4,5)^ and improving nutrition knowledge^(5)^. Despite evidence for a small but positive impact of nutrition education on children, researchers suggest that effectiveness could be enhanced by addressing issues related to programme implementation^(5–7)^.

Implementation of school-based nutrition interventions largely depends on barriers and facilitators experienced by teachers^(8,9)^. Teacher characteristics, such as previous experience/knowledge, motivation and interest, as well as comprehensive educational material, teaching training and support from programme staff contribute to enhanced implementation of nutrition curricula^(9–11)^. Furthermore, many studies have investigated barriers and consistently found that particularly limited time and competing demands hinder teaching of nutrition^(8,12–20)^. Therefore, the use of cross-curricular or integrative teaching strategies has been proposed as a solution to combat these barriers^(14,16,18,19,21)^. Embedding nutrition within core subjects could support the uptake of nutrition education without reducing the time spent on mandatory subjects^(14,22)^. Additionally, nutrition concepts could provide a real-life context that enhances the learning of other core subjects^(9,23,24)^. Although this approach has gained some interest, it remains unknown whether it addresses teachers’ time constraints and how teachers and students perceive this teaching strategy^(25)^.

A recent scoping review concluded that more research is needed to explore the effectiveness of cross-curricular approaches^(25)^. These future trials require comprehensive process evaluations to understand why some interventions succeed while others fail^(26)^. Many studies use quantitative data to examine intervention fidelity and acceptability, while only few provide insights from qualitative data^(26)^. Not surprisingly, previous research warrants the need for rigorous process evaluations including both quantitative and qualitative outcomes^(6,25–27)^.

The Cross-curricular Unit on Portion Size (CUPS) program was evaluated using a pilot cluster randomised controlled trial (RCT) in Australian primary schools. CUPS was designed to improve children’s portion size estimation skills and nutrition knowledge while using an integrative teaching approach^(28)^. The CUPS intervention comprised six lessons on nutrition and mathematics concepts for Stage 2 students (aged 8–10 years). Teachers received training and resources to implement the programme into their classrooms across 1–4 weeks. The programme proved effective towards students’ nutrition knowledge scores (*P* < 0·01)^(29)^, and whilst non-significant due to the sample size not being large enough to provide sufficient statistical power, a slight improvement in portion size estimation skills was observed among children receiving the programme in comparison to a control condition (Follong *et al*., unpublished results).

The current paper describes the findings from the process evaluation, embedded within a cluster RCT examining the programme effectiveness, to identify teachers’ and students’ experiences with the programme and the use of an integrative teaching strategy. This process evaluation goes beyond examining programme acceptability and explores barriers and facilitators that affected its implementation and effectiveness. We sought to distinguish which aspects of the intervention were successful and which elements would need amendments to guide future studies using a similar teaching strategy.

## Methods

The CUPS intervention followed a mixed method design. It consisted of a cluster RCT designed to investigate the effectiveness of integrative nutrition education for primary school children and qualitative data collection embedded within the trial to explore teachers’ and students’ perceptions of the educational programme and teaching approach. The trial was designed according to the Consolidated Standards of Reporting Trials and registered with the Australian and New Zealand Clinical Trial Registry (ACTRN12619001071112). A recent publication describes the intervention and methods used to conduct the research in detail^(28)^. Student nutrition knowledge (survey) and portion size estimation skills (estimation task) were assessed at baseline, immediately post-intervention and 4 weeks later as part of the RCT component. To complement the quantitative student data obtained during the RCT, interviews and focus groups were held with participating teachers and students, respectively. Only qualitative data are reported in this paper.

### Participants

Four primary schools (5 Year 3 and/or 4 teachers) from the Newcastle and Hunter region (New South Wales (NSW), Australia) participated in the RCT. Teachers (*n* 3) randomised to the intervention condition and a selection of their students were involved in the current evaluation study. Voluntary consent was sought from all participants. On average, teachers had 9 years of experience in teaching a variety of primary school levels. None of the teachers had prior experience in teaching nutrition, nor were they trained in any specialist areas. A mix (e.g. gender, age and achievement level) of five students were selected by their teachers to participate in the evaluation. This ensured the selected students represented the wider student population involved in the CUPS program.

### Intervention

The CUPS intervention involved the implementation of an integrated teaching unit that targeted student learning on both nutrition and mathematics concepts such as appropriate portion sizes per food group and volume and capacity. Experienced primary school teachers and experts in the field of Nutrition research collaborated to develop the final six lesson plans. The NSW K-10 syllabus for Mathematics and Personal Development, Health and Physical Education, and materials on the Australian Guide to Healthy Eating recommendations were used to create the content. A recent protocol paper outlines the CUPS lesson content, sequence and learning outcomes^(28)^. In contrast to what was stated in the protocol, content was spread over six instead of the five lessons. Lesson two was split into two identical lessons except for the example foods used to measure portion size (i.e. example foods from the five food groups were divided over the two lessons instead of all examples being discussed during one lesson). This ensured students had adequate exposure to the content across two lessons. The researchers believed students would need more time and practice to get a grasp of portion size estimations given that a previous study with children concluded that they may need multiple lessons^(30)^.

Before programme implementation occurred, teachers attended a 3-h professional development workshop delivered by the research team. The aim and content of this workshop are described elsewhere^(28)^. Briefly, teachers were taught how to prepare, plan and implement the CUPS lessons. The relevance and rationale for the integrative and experiential approach was discussed. Moreover, teachers were familiarised with content on healthy eating guidelines and portion size estimation, with the majority of the time focussing on how to integrate this content into the Stage 2 Mathematics curriculum. Following completion of the workshop, teachers were given a CUPS school pack containing all resources (i.e. mathematics cubes, food models, measuring cups, plastic containers, Australian Guide to Healthy Eating brochures and posters, lesson plans, worksheets and presentation slides). Teachers completed six pre-planned lessons of each approximately 40 min in duration, which were taught across 3 to 4 weeks (approximately two lessons/week).

### Evaluation outcomes

The acceptability, experiences and potential of the CUPS program were examined in the process evaluation using two qualitative research methods. Semi-structured interviews and focus groups were conducted to explore both teachers’ and students’ perspectives on the intervention. The full research method protocol including all interview and focus group questions has been recently published^(28)^. Questions were adapted from a previous physical activity programme in schools using a similar methodology^(31)^.

Interviews focusing on perceptions, facilitators and barriers of the programme implementation and delivery, and content and resources were conducted by phone shortly after finishing the last lesson. Teachers were asked about their experiences with the teaching unit in comparison with regular volume and capacity lessons. Additionally, 20-min focus groups were held with the selected students within 1 week of completing the intervention at their schools. Questions were designed to explore students’ thoughts on the CUPS lessons in comparison with their usual mathematics classes, as well as their opinion on enjoyment regarding both classroom activities and materials, learning outcomes and further improvements of the programme. Furthermore, teachers and students were asked to rate their agreement with several statements using a five-point Likert-type scale (high scores representing high agreement). The interviews and focus groups were audio recorded and transcribed verbatim by a secure transcription service.

### Data analysis

Data were analysed in line with Creswell’s (2014) six steps for qualitative analysis by the lead author^(32)^. Audio recordings were transcribed, and digital transcripts were subsequently managed using NVivo software (Version 12). First, all transcripts were repeatedly reviewed to gain a general sense of the data. Themes were inductively identified and defined by a process of sequential revision, refinement and coding from the raw interview and focus group data^(33)^. The lead author generated a list of themes, followed by the identification of subthemes. The next step involved interpreting the significance of these themes. Lastly, quotes that corresponded with the (sub-)themes were extracted from the transcripts. Average scores for statement agreement were calculated.

## Results

### Teacher interviews

Five themes were identified that highlight the teachers’ experiences and perceptions on the CUPS program. The themes are described with example quotes below and in Table 1.

**Table 1:** Themes and subthemes including illustrative quotes that emerged from teacher interviews on the CUPS program (*n* 3)

Themes and subtheme description	Quotes
*CUPS lesson content*	
The overall lesson content, sequence and integrative concept was well received	‘The lessons themselves were really good. […] It was really informing and I think the kids enjoyed it as well. Yeah, it was a good concept. It just needed a little bit of tweaking with those lessons, I think. That’s all.’ Teacher 2 ‘I think it’s great to bring in the maths, because if we were just teaching nutrition, we’d just be doing a lot of talking and a lot of looking at pictures. […] Yeah, it made it very practical, so I think it sunk in more.’ Teacher 3
Nutrition content was perceived as really good	‘It was definitely more nutrition based […] So yeah, it had good knowledge about the nutrition side of things.’ Teacher 2
Lacking content and depth on volume and capacity concept	‘However, with the volume and capacity, I just don’t know how much it did link to volume. It was definitely a nutrition lesson but […] I don’t know how closely connected it was to volume and what the kids need to know about volume.’ Teacher 2
Does not fully replace usual lessons on volume and capacity	‘I don’t know if it could replace it completely, but I think if a few things are maybe just changed a little bit, yes, I think so. […] and then if we just get a bit more in-depth about the cups, millilitres and grams of the different foods, then I think it could be.’ Teacher 1
Includes a variety of mathematics concepts	‘Yes, they definitely built their maths skills too. It was just they were building a variety of maths skills. […] They were doing fractions, they were doing multiplying, they were doing estimating, and they were doing a little bit of volume. They’re all good things. […] Yeah, it was very cross-curricular in terms of tapping into other aspects of maths, rather than just being about volume.’ Teacher 3
*CUPS resources and materials*	
The materials such as the lesson plans, worksheets and PowerPoint Presentations were perceived as very detailed and easy-to-use	‘I thought the lessons were very detailed and all the worksheets were great, and the PowerPoints were really easy to explain. Yeah, so I thought the rest was quite good.’ Teacher 1
The food models, mathematics cubes and measuring cups were great hands-on tools to engage the students and visualise the concepts taught	‘Well they were very engaged when the food and the cubes were out, yeah very hands-on which was good because my kids really liked that.’ Teacher 1 ‘It’s just good to hold it. I think the more physical it can be, where they’re holding it in their hands - I mean, it is a very physical program’ Teacher 3
*Student learning and achievement*	
Students had a better understanding of the nutrition concepts, and they were able to put the knowledge and skills gained into practice	‘Then even just understanding what might be a healthier option than something else, like when they were doing that the follow-up assessment, I noticed that I think they understood that a bit better, so that benefitted their results.’ Teacher 1 ‘I think - well it will be interesting to see, but I think the kids will retain a lot about nutrition, and estimating, that they’ll carry with them into their real lives.’ Teacher 3
Some students might have understood the estimations and mathematical conversions, whereas others did not as a few concepts might have been confusing	‘I think they understood, once we went through it, the cubes, and how it matched up to the serve sizes and they definitely got better as the lessons went on. I think they were a little bit confused by that at the start, but they got better at that, I thought.’ Teacher 1
The majority of the students enjoyed the lessons	‘Yes, I think most of them did enjoy most of the lessons. I think at times there’s always students that get a bit distracted or aren’t paying attention the whole time. But I think they were mostly engaged and I think having the fake foods there, and the cubes, they liked playing with the cubes. So I think they were mostly engaged and enjoying it.’ Teacher 1 ‘I think some of them found it a little bit repetitive but I’d say the majority of them did enjoy it.’ Teacher 2
Students were engaged most to the time, and they might have had a greater engagement as a result of the nutrition integration compared with their usual maths lessons	“For the most part, the kids totally engaged and learnt from each other, as well as from me.” Teacher 3 ‘Oh, yeah [I think the nutrition integration contributed to a greater engagement compared to our usual maths lessons]. I would say anything integrated because it’s something a little bit different than the norm is exciting and different. I did think that that they did benefit in it having that integration into it.’ Teacher 2
*Teaching experience*	
The professional development was informative and very valuable	‘Yes, I did enjoy that. It really opened up my eyes to nutrition in general. […] I found it really valuable. To be able to see a lesson being taught as well was really good for me to be able to go in and then teach my own class. Yeah, I thought it was quite valuable and good information.’ Teacher 2
Teachers enjoyed teaching the CUPS lessons	‘I enjoyed teaching the lessons because it was something different.’ Teacher 2 “It is always enjoyable to do those subjects that are hands-on.’ Teacher 3
Teachers felt moderately confident about teaching the CUPS lessons mostly because of having limited knowledge about nutrition	‘Yeah, I think I was [confident]. Once I’d done a little bit of research myself […] like as you do with all lessons I always like to go and have a look at the lesson and see what the content is and all that kind of stuff before I teach it. Once I had actually, I guess you could say, do a mini research myself before I taught the lesson, I was confident…’ Teacher 2
Teachers expressed that they would like to use more integrative strategies in their future teaching, and that it is important to incorporate nutrition into the curriculum	‘I believe in cross-curricular integration. At this day in age you need to do cross-curricular as you don’t fit a lot of the stuff in. […] In terms of time constraints it needs to be integrated. It’s unfortunate that it can’t be its own program. […] I would [like to use cross-curricular teaching], you just need to plan for it.’ Teacher 2
*Programme benefits and challenges*	
Teachers indicated that one of the major benefits of the programme was that they were able to teach their students about nutrition	‘Well as I said we don’t get to teach nutrition that much, so to go over that was really good. I think the kids need to be aware of it, of what they should be eating and how much they should be eating, because some of them just have no idea that there’s even guidelines for that. I think that was good.’ Teacher 1 ‘I think just the fact of being able to teach nutrition to the students because I haven’t. […] I think that was probably the benefits that I had out of it…’ Teacher 2
Teachers observed that the students benefitted from by programme as their nutrition knowledge improved	‘I found that a lot of students actually didn’t know [about the food groups or that you weren’t supposed to just have a balanced meal] so it was good to see the benefits of the students actually understanding.’ Teacher 2
Not enough resources were available for all children to participate in the activities at the same time	‘Yeah, I think that was probably just my main challenge was just that, was just like the resources in terms of trying to get the kids to, all of them, to participate and not just my children who were super involved in it. Not just getting them to take over.’ Teacher 2 ‘I think that we probably need a few more resources.’ Teacher 3
Not being able to differentiate the lessons for students at varying levels	‘There was only that one lesson that I stressed out a little bit about which had integrated a lot of the maths concepts. The reason I did stress out a bit at that was because the kids in my class were quite differentiated, if you know what I mean. I had children who were working really low who would not understand that concept and then I had children who were working quite high that would understand that concept.’ Teacher 2 ‘Varying student knowledge, I guess, is sometimes an issue.’ Teacher 3

#### Cross-curricular Unit on Portion Size lesson content

In general, the lesson content was perceived as really good by all the teachers. Each teacher highlighted aspects that they thought were good and aspects that could use improvements. According to all teachers, the lesson content sufficiently supported the learning of nutrition concepts but missed depth on volume and capacity. As a result, two teachers commented that they believed the CUPS lessons could not fully replace their usual mathematics lessons on these concepts. The following quotes illustrate these three findings:

‘I think 100 % [supports the learning of] nutrition [concepts]. If you wanted to tackle nutrition, that’s the way to do it.’ Teacher 3

‘It definitely complements the mathematics of volume and capacity, but it does lend itself more so, I’d say, to a health lesson.’ Teacher 3

‘I guess it’s so different to anything else in the maths syllabus, because you’ve combined the two [subjects]. It steers quite far away from your traditional way to teach volume and capacity. So, you couldn’t really say it would be a replacement to volume and capacity as a maths lesson.’ Teacher 2

Although the lessons might have not contained in-depth volume and capacity concepts, the lessons ‘*tapped into other aspects of maths, rather than just being about volume*’ (Teacher 3). Teachers reported students learning about estimating, problem solving, reading tables, fractions, multiplying and adding multiples.

#### Cross-curricular Unit on Portion Size resources and materials

The CUPS resources and materials were of great value to the teachers. The ‘*ready-to-go*’ materials were ‘*really appealing*’ (Teacher 3), easy to use and very detailed. The lesson plans were good to refer to during the lessons and therefore supportive of teaching.

‘The lessons were really thorough. You couldn’t say you didn’t have enough information. The slides were really clear, but not over the top. […] I can pay for a subscription to a resource, and not get the level of detail.’ Teacher 3

In terms of the food models and mathematics cubes, two teachers commented that these resources were great hands-on tools that were engaging for the students and helped visualising the food volumes:

‘Yeah it was good to have it, to actually have all those fake foods for the kids to see, because I think a lot of kids need that visualising of the actual food as well; instead of just telling them what a serve size is, when they see it they’ll be able to better understand it and remember it. So yes, I thought that was good.’ Teacher 1

#### Student learning and achievement

Students clearly showed improvements in their nutrition knowledge according to all teachers. Teachers stated that the students have a better understanding of the food groups, serve sizes and the number of serves they are allowed to have, healthy and unhealthy foods and balanced meals. This sparked students’ interest to apply their knowledge outside of the classroom:

‘…I feel like they were really starting to get it and they were linking it to real life. In the playground they were looking at the nutrition patterns on the back of their foods and stuff like that.’ Teacher 2

A major part of the mathematical content of the CUPS lessons involved estimating volumes and the conversion of cubes to cups and vice versa. All teachers commented on their students’ ability or understanding of these mathematical concepts which showed that this varied widely. Some children struggled with visualisations of squared cubes fitting into round cups, whereas others found it hard to conceptualise estimating volumes of a group of food shapes. Others might have memorised the conversions rather than understanding the mechanism behind it. The quotes below demonstrate the large differences in student understanding:

‘The estimating skills hugely improved by using the CUPS cubes.’ Teacher 3

‘I think mathematically that’s a hard concept to look at it in terms for the Year 4 students and Year 3 students. […] Once they worked out [a pattern] then they were okay with it, but I think they would not have probably understood that mathematical conversion of how to work it out unless they just memorised it.’ Teacher 2

Overall, many students enjoyed the CUPS lessons and the majority were engaged (score 3·3/5·0). Two out of three teachers also mentioned their students showed greater engagement compared with their usual mathematics lessons as a result of the nutrition integration being ‘*different than the norm*’ (Teacher 2) and ‘*tangible*’ (Teacher 3). However, these two teachers also briefly stated that the lessons may have been somewhat repetitive for a few of their students.

‘Yes, definitely [nutrition integration contributed to a greater engagement compared to our usual maths lessons]. Because it was tangible, and it was real.’ Teacher 3

#### Teaching experience

The professional development workshop was informative, valuable and ‘*a great starting point*’ (Teacher 3).

‘Yes, I thought it was very informative and it helped me to understand what I was going to be teaching the kids.’ Teacher 1

All teachers enjoyed teaching the lessons (score 4·0/5·0), and they felt moderately confident while doing this (score 3·7/5·0). They were less confident about their nutrition background knowledge:

‘My confidence grew obviously, and as I got more and more into it, as did the kids. Yes, I felt pretty confident. I was caught out a couple of times, but it was mainly about nutrition questions I couldn’t answer.’ Teacher 3

After teaching the CUPS lessons, each of the teachers expressed they would like to continue using an integrative approach to teach nutrition.

‘I would definitely like to [continue using an integrated approach to teach nutrition]. Sometimes it can be tricky, but if I was given the opportunity to, I would. It makes it a bit easier and probably more interesting for the kids as well.’ Teacher 1

#### Programme benefits and challenges

All teachers agreed that being able to teach nutrition to their students was a major benefit of the programme. They expressed that they usually do not get to teach nutrition often. Another benefit was that the teachers noticed significant improvements in students’ nutrition knowledge.

‘I think, yeah probably the best thing was that I could link nutrition into the classroom because I really haven’t done that at all this year with this class. So actually teaching them about something that I am, I guess, quite passionate about as well was good.’ Teacher 1

‘Definitely an understanding of what is in each food group, and the number of serves that they can have of each food group in a day, I think that point got across really well. I think that they have a better understanding of just everyday healthy food, *v*. discretionary, sometimes food.’ Teacher 3

In contrast, the teachers experienced several challenges to teaching the lessons such as limited resources and not being able to differentiate the lessons. Students might have not been able to participate due to groups being too large and a few teachers felt they were not always able to accommodate varying student knowledge as some lessons did not allow for differentiation. The following quotes substantiate these challenges, respectively:

‘I think maybe just when we had to measure, the kids had to be in groups and they were estimating […]. I kind of found there probably wasn’t enough of the fake foods for the groups were so big, so I’d found some kids just kind of sitting there, not doing anything, and that’s when I think they got a bit distracted.’ Teacher 1

‘I think it was more like I was trying to work out a way that I could differentiate it so I could make it easier for most of my class to be able to answer that […]. I found that was a little bit tricky because I could tell for some of my kids it was just going over their heads. Because that lesson was only one lesson, usually if it was something like that, a concept that I knew the kids needed to know but I couldn’t really differentiate it, then I would probably teach it over a couple of lessons. But other than that, I think the kids really got it in the end.’ Teacher 2

### Student focus groups

Focus groups were held with five students from each class including both boys and girls (*n* 15). Student answers were classified into three themes that are summarised in Table 2.

**Table 2:** Themes and subthemes including illustrative quotes that emerged from student focus groups on the CUPS program (*n* 15)

Theme and subtheme description	Quote
*Programme enjoyment*	
All students enjoyed the CUPS lessons	‘It was fun. It was nice learning something new.’ Student, class 3
Student opinions varied on the activities that they enjoyed the most and the least	‘I liked the sugar one where we had to figure out which had more sugar.’ Student, class 1 ‘All of [the activities]. Especially the lunchbox one.’ Student, class 3 ‘[Lesson five] was confusing.’ Student, class 2
The repetitiveness of the lessons and group work were perceived as less enjoyable by a few students	‘My least favourite was the one where you had to estimate the cubes because once we did it a few times it kind of got a little boring.’ Student, class 1
The best things about being involved were the fun lessons and learning about nutrition	‘You get to do fun lessons.’ Student, class 1 ‘That I learned about healthy eating.’ Student, class 2
*CUPS resources and materials*	
Almost all students enjoyed using the food models and mathematics cubes	‘I liked the cubes, but they kept falling apart.’ Student, class 2 ‘[The food models] were so realistic.’ Student, class 3
Using food models and mathematics cubes made the activities more interesting	‘It makes it less confusing. […] because you don’t have to guess it.’ Student, class 2
Lessons do not need many improvements except for the instructions and group work	‘Just maybe more instructions.’ Student, class 2 ‘In smaller groups.’ Student, class 3
*Student learning and achievement*	
The real-life nutrition context helped some students learn about mathematics	‘Yes, estimation you mainly use maths.’ Student, class 1
Students gained knowledge on healthy eating and the guidelines	‘[I now know] how much of serves we’re supposed to have a day and that.’ Student, class 1

#### Programme enjoyment

All students reported that they enjoyed the CUPS lessons with a variety of examples or reasons given (score 4·4/5·0). Students stated they enjoyed the programme because it could ‘*help with your health*’, ‘*it was challenging*’ and ‘*it was fun and nice learning something new*’ (Students, class 3).

‘I enjoyed them because they were fun.’ Student, class 1

Answers to the question ‘What kind of activities did you enjoy doing in the CUPS program?’ varied greatly. Students referred to liking specific lessons such as the lessons on food groups and serve sizes (*n* 1), serve size estimations (*n* 2), sugar volume (*n* 3), creating your own lunchbox (*n* 6) and all lessons (*n* 2). Furthermore, some students indicated that they enjoyed completing the assessments as part of the effectiveness trial at the start and end of the programme (*n* 4).

Whereas two students enjoyed the serve size estimation activities the most, others expressed they did not enjoy these lessons (*n* 8). Three students highlighted that these lessons were repetitive and therefore less enjoyable. Other than being repetitive, these three lessons also involved a lot of group work. A few students found it difficult or frustrating to work in such large groups sharing only limited food models:

‘Well, maybe not so much the ones where we had to go around in the groups. Maybe it was just some of the people in my group weren’t cooperating well. […] Because some of the people on my group weren’t letting any other people have a turn of like giving them what their opinion of it and what they think of it, so it’s a bit frustrating.’ Student, class 3

Furthermore, four children stated that they ‘*liked them all*’ (Students, class 1 and class 2) or that there was ‘*nothing*’ (Student, class 3) they did not enjoy. Similar to the teachers, students thought the best thing about being involved in the programme was learning about healthy eating in a fun way.

#### Cross-curricular Unit on Portion Size resources and materials

Other positive aspects of the programme were the resources and materials. Except for two students, all children stated that they enjoyed using the food models in the classroom. The food models were perceived as fun and looking like real foods. The reason for not enjoying the food models was the fact that they were not edible. In addition, almost all students liked using the cubes in the lessons as they were ‘*cool*’ (Student, class 1), ‘*fun*’ (Students, class 1) and ‘*colourful*’ (Student, class 3). About one-third of the children commented that the cubes kept falling apart, which was perceived as annoying by some of these children.

‘Yes [I liked the cubes], it can help combine health and maths together.’ Student, class 1

The use of food models and cubes resulted in the mathematics activities being reported as more interesting for the students (score 4·1/5·0). Children from one class in particular said that the food models could be used as visual examples.

Although many students said nothing needed to change in the programme, two students suggested the instructions and group work could be improved by simplifying the instructions and decreasing the number of students to collaborate with.

#### Student learning and achievement

The last theme relates to student understanding, learning and achievements. All students were able to tell that the CUPS program was about healthy eating, food groups and serve sizes. However, only some may have understood that this content was linked to mathematics.

The majority of the students thought that the real-life nutrition topics helped them learn. In two focus groups (classes 1 and 3), all students reported that the nutrition topics supported their mathematics learning in regard to ‘*volume and capacity*’, ‘*measuring with cubes*’ and ‘*estimating*’.

‘Yes, estimation you mainly use maths.’ Student, class 1

In one focus group (class 2), the students felt that the real-life context did ‘*not really*’ help them learn. According to the students in this group, the lessons supported the learning of some mathematical concepts including counting and multiplications and that the lessons ‘*enforced more nutrition than maths*’.

After receiving the CUPS lessons, all students believed that they had improved their knowledge on nutrition and healthy eating. Examples given by the children on topics that they know more about compared with before the CUPS program included the food groups, guidelines on serve sizes, number of recommended serves and differences between gender and age:

‘I didn’t know this before that girls actually have to eat more dairy and boys actually have to eat more grains.’ Student, class 3

## Discussion

The current study evaluated experiences and acceptability of the CUPS program that involved teaching about portion size and mathematics in primary schools. The intervention used an integrative approach and experiential learning to teach students about healthy eating, serve sizes and portion size estimations while embedding educational standards from the NSW Mathematics syllabus on unit of measurement. The programme was generally well received by both teachers and students, with programme-specific challenges suggesting future improvements.

Results from the interviews and focus groups revealed that the programme acceptance was relatively high. Teachers and students reported enjoying teaching and receiving the lessons, respectively. In particular, the nutrition content on healthy eating, food groups and serve size recommendations was liked and the ability to learn about these topics was valued the most. Teachers and students stated that they noticed considerable improvements in nutrition knowledge and students used the gained knowledge outside of the classroom. These positive attitudes and observations are in line with our findings of the programme’s impact on student nutrition knowledge scores which significantly increased for students receiving the lessons compared with students who did not (Follong *et al*., unpublished results). This clearly shows that the CUPS program could contribute to enjoyable and effective nutrition education within the primary school setting.

Student enjoyment and engagement could be further enhanced by small amendments to the teaching unit and intervention design that would result in less repetitiveness and improved group work. Lesson content was purposefully designed to be somewhat repetitive and to some extent overlap. Reasoning for this was that previous research suggested that to improve estimation accuracy, children needed more than one training session^(30)^. Strategies to refine the programme could be to reduce the number of lessons that involve portion size estimations by consolidating lessons. Two lessons contained the same activities but only differed in the food models used. This would also decrease the number of students per food item and thereby improve the group work that was reported as troublesome. In addition, spreading the lessons across a longer intervention period might diminish the perception of repetition. It is therefore recommended to take into account the intervention design in terms of the intervention length and spread of the lessons when developing new educational programmes to ensure effective programme implementation and optimal student engagement.

Connecting real-life nutrition topics to abstract mathematics concepts has been suggested to enhance student engagement and academic achievement^(23,34–36)^. In the present study, teachers expressed that students were more engaged with the lessons due to its integrative nature, and some students thought that the real-life nutrition context helped them learn about mathematics. Furthermore, the use of both food models and mathematics cubes made the lessons more interesting for the students. However, teachers noted that the lessons steered quite far away from their typical teaching, and as highlighted in both interview and focus group data, most students did not identify the mathematics content in the lessons. While the connection between nutrition and mathematics was explicit within the lesson plans and activities, integration might needs to be made more explicit to the students in order for them to recognise the relevance of mathematics in real-life settings and to produce meaningful connections^(37)^. Therefore, it is essential that educational programmes using a similar approach incorporate teacher instructions and classroom activities that focus on the rationale an importance of the curricular integration. Future trials should assess student mathematics achievement to examine the impact of the programme in comparison with traditional mathematics lessons.

The integrative lessons were designed to incorporate learning outcomes from the NSW Mathematics syllabus that target volume and capacity. Despite the lessons involving learning of various mathematical concepts, teachers would prefer more in-depth content on volume and capacity. The learning of volume and capacity was perceived as very different to their usual teaching and therefore did not fully match the syllabus. Consequently, teachers indicated the CUPS lessons could not replace their usual teaching unit on this mathematical topic and might therefore add additional time to their teaching schedule^(10)^. It can thus not be assumed that any form of integration with core subjects would be beneficial for teachers’ classroom time. These findings highlight the complexity of integrating two or more subjects and the need for a seamless fit with the teachers’ standard teaching practices. Although previous research^(18,19)^ and the current study show that teachers believe curricular integration may be an effective approach to enhance the implementation of nutrition education in schools, it remains unclear what teachers expectations, preferences and needs are towards using this teaching strategy. While the current study involved nutrition education that integrates mathematics concepts, teachers might prefer mathematics lessons that incorporate nutrition examples. Future research should therefore investigate teachers’ preferences and needs and explore the most suitable and effective way to use an integrative teaching approach.

Another concern that arises as a result of the limited mathematics content is the programme’s impact on student learning and understanding of portion size estimations. To accurately estimate food portions, knowledge on mathematical concepts such as volume and capacity is essential^(38)^. Students’ ability to understand the concepts behind portion size estimation might have been hindered by the insufficient link with volume and capacity. This was further supported by teacher’ comments on confusing content. Some students might have understood the concepts taught, whereas others found it difficult to comprehend. A reason for this might be that not all teachers were able to differentiate some lessons and therefore could not adjust for varying student abilities. There was no guidance provided in the professional development workshop and written materials for teachers to adapt or differentiate the lessons. A process evaluation of the Active for Life Year 5 program on diet and physical activity reported similar feedback from teachers, where some teachers expressed that the lessons did not always fit their students learning abilities^(27)^. As with this previous programme, amendments should be made to the workshop and CUPS lessons to account for different levels of ability, particularly on the mathematics content^(27)^. Future trials might need to consider developing educational resources together with participating teachers as this potentially increases feasibility, uptake and alignment with traditional teaching content^(18,27)^, especially when using an integrative approach^(25)^. This way, teachers would also be able to ensure that the programme accommodates their students’ abilities.

Additionally, the limited number of resources was suggested to influence student learning. Adequate provision of resources is paramount for successful implementation of experiential nutrition programmes^(21)^. Some students might have simply not been able to participate in the activities as groups were too large to all work with the food models and cubes at the same time. As stated earlier, supplying a sufficient amount of resources would also contribute to higher student engagement and enjoyment by allowing the teachers to divide the classroom into smaller groups. This addresses the students’ comments on programme improvements and should be taken into account in future trials to enhance programme implementation and effectiveness.

Students enjoyed using the hands-on tools (e.g. food models and mathematics cubes) to estimate portion size. According to both teachers and students, these physical resources were particularly useful as visual reference when learning about appropriate serve sizes. Food replicas are frequently used in nutrition and portion size research^(39,40)^. Food models have several benefits over real food that make them practical for use in the educational setting. Food models do not require preparation and food waste is avoided by repeatedly using the same models^(40)^. Except for some of the cubes breaking apart while handling them, they were perceived as a fun tool to combine nutrition and mathematics. Mathematics cubes are commonly used in the classroom to teach children about volume and capacity^(38)^. These cubes have also been tested as a new portion size estimation aid in the adult population, with results showing improved accuracy compared with other aids^(41)^. Although the cubes were fun and familiar to the students, the intervention was not able to replicate these previous observations. In comparison with students not receiving the CUPS intervention, students did not significantly improve estimation accuracy of food portions (Follong *et al.*, unpublished results). Age-appropriateness of the cubes as a portion size estimation aid for primary school children should be further explored.

After completing the professional development workshop, teachers felt relatively confident about teaching the unit. Their level of confidence was primarily affected by their restricted nutrition knowledge, which required some teachers to further research the content in preparation of the lessons. Similar findings were observed for a school-based diet and physical activity intervention in the United Kingdom in which teachers indicated that for almost half of the lessons more preparation time than usual was required^(27)^. The extra time and effort associated with lessons involving new concepts and strategies might diminish over time when teachers become more familiar with it^(31)^. However, the intervention length and number of lessons might have not provided the teachers with enough time and practice to do so and they may have benefitted from regular contact with the researchers throughout the intervention period. Furthermore, adequate teacher training is an important factor to successfully implement nutrition education^(42)^. Training has proven to improve teachers’ self-efficacy and may thus lead to more effective nutrition education^(43)^ and better programme implementation^(44)^. The FoodMASTER program used a similar approach to integrate nutrition, mathematics and science in American primary schools. Findings showed that teachers involved in a 1-d training significantly increased their self-efficacy towards teaching nutrition, with largest improvements observed for teachers’ understanding of nutrition concepts^(43)^. A recent systematic review concluded that it is critical for teacher training to focus on nutrition content and behaviour change techniques, with some research suggesting a minimum of 20 h being ideal^(45)^. Therefore, expanding the duration and nutrition content of the CUPS professional development workshop might be necessary to adequately support teachers implementing the programme and ultimately facilitating student learning.

### Strength and limitations

Limitations should be considered when interpreting the data. With three teachers implementing the intervention, the sample size of the current study was small. The number of interviews and focus group conducted was therefore low and saturation might have not been obtained. Another limitation might be that both teachers and students may have given socially desirable answers. Furthermore, all teachers had an interest in nutrition and thought nutrition education was important to support their students’ health. This might have influenced the teachers’ motivation to provide the lessons and may have impacted their interview answers. Verification of findings by a larger group of participants that better represents the wider teacher and student population is essential. Although all lessons were delivered to the students by trained teachers, intervention fidelity was not examined. Potential differences in the implementation of the programme between classes might have influenced the teacher’ and student’ experiences and therefore could have impacted the findings of this evaluation study. Lastly, data analysis was performed by a single researcher, and codes were not checked by the other authors.

The current study describes qualitative data exploring both teachers’ and students’ experiences with the programme. An in-depth evaluation of the programme was performed that highlights the areas that need improvement not only for the CUPS intervention but also for nutrition education programmes in general. Taking into account the perspectives of not only the teachers but also the students ensured that programme enhancement benefits both parties. Since the need for qualitative research on integrative nutrition education is warranted^(25)^, our observations provide novel insights that inform future research about integrating nutrition on what works well and what aspects require further investigation.

## Conclusions

Our findings provide insight into major challenges and successes of implementing an integrative nutrition programme, its effect on student learning and enjoyment and suggestions for improvements. The current study is one of only a few reporting on the impact of integrative teaching on teachers’ time barriers to implement nutrition education. Due to insufficient level of depth on specific mathematical concepts and teachers’ lack of nutrition knowledge, this programme might have not reduced teachers’ time constraints. This, together with difficulties differentiating for student’ ability and limited resources, may have impacted on the effectiveness of the intervention to demonstrate improved estimation accuracy. Nevertheless, teachers and students experienced the programme as enjoyable and valuable specifically in regard to nutrition. To build a more complete picture of cross-curricular teaching, a better understanding of teachers’ preferences and needs towards nutrition integration into the primary school curriculum is fundamental. It is recommended that researchers work closely together with participating teachers to develop effective nutrition lessons that are embedded into other school subjects.
